# Colonic basidiobolomycosis in a patient with systemic lupus erythematosus (SLE)

**DOI:** 10.1186/s12879-022-07720-9

**Published:** 2022-09-16

**Authors:** Elham Barahimi, Tuba Abbasi, Zahra Ghaeini Hesarooeyeh, Hanieh Raad, Mohadeseh Karimi, Mahsa Shahi

**Affiliations:** 1 Infectious and Tropical Diseases Research Center, Hormozgan Health Institute, Hormozgan University of Medical Sciences, Bandar Abbas, Iran; 2 Department of Pathology, Faculty of Medicine, Hormozgan University of Medical Sciences, Bandar Abbas, Iran; 3 Student Research Committee, Faculty of Medicine, Hormozgan University of Medical Sciences, Bandar Abbas, Iran; 4 Student Research Committee, Faculty of Paramedicine, Hormozgan University of Medical Sciences, Bandar Abbas, Iran

**Keywords:** Gastrointestinal basidiobolomycosis, Fungal infection, Abdominal mass, Systemic lupus erythematosus, SLE, Basidiobolus ranarum, Pathology

## Abstract

**Background:**

Basidiobolus ranarum belongs to the Entomophthorales order and the Zygomycetes class. This fungus is an environmental saprophyte that can be found in soil and rotting vegetables.Primarily restricted to tropical regions including Asia, Africa, and South America. It might cause chronic inflammatory diseases, mostly affect subcutaneous tissue. Systemic infections involving the gastrointestinal tract are extremely rare.

**Case presentation:**

Herein, we present a 44-year-old Persian man with the past medical history of lupus erythematosus with colicky abdominal pain started from three months before admission with many vomiting episodes, and a mass on the right lower quadrant, who had been thought initially to have an abdominal malignancy. The patient had vital signs were within normal ranges. His physical examination revealed tenderness and rebound tenderness on the right lower quadrant of the abdomen. A fixed mass 10 × 10 centimeter in diameter was palpated in the same quadrant. Laboratory, radiologic, colonoscopic examination was requested. The patient underwent laparotomy which revealed a mass in the terminal ileum and ascending colon with retroperitoneal adhesion and invasion to the right ureter behind it. Pathologic examination showed basidiobolomycosis infection in the specimen.

**Conclusion:**

Fungal infection should be among the differential diagnoses for adults present with abdominal mass in endemic regions of the world.

## Background

Basidiobolomycosis is a rare disease caused by the fungus named Basidiobolus ranarum, which could be found all over the world. B. ranarum belongs to the Entomophthorales family and the Zygomycetes class [1]. It was first described in 1886 in frogs. This fungus is an environmental saprophyte that can be found in soil and rotting vegetables. Primarily restricted to the tropical regions including Asia, Africa, and South America [2–5].

Basidiobolomycosis, a chronic inflammatory disease, can range from mild subcutaneous lesions involving the buttocks, trunk, and limbs to systemic infections involving the gastrointestinal (GI) tract; however, extracutaneous involvement is extremely rare [1, 6, 7]. Gastrointestinal basidiobolomycosis (GIB) has been reported due to ingestion of soil, animal feces, and contaminated food [8–10].

Systemic lupus erythematosus(SLE) is a chronic systemic autoimmune disease. There are different regimens for maintenance therapy of the disease. Corticosteroids play a key role in control of SLE patients’ symptoms. As previous studies showed, there is a relationship between steroid use and incident of fungal infection [11].

This report describes the GI Basidiobolus ranarum infection in an adult with SLE disease from southern Iran.

## Case presentation

The patient was a 44-year-old man with past medical history of lupus erythematosus for more than eight years. He was a farmer, living in Hormozgan province, in south Iran, with the chief complaint of abdominal pain. His pain was colicky on the right lower quadrant started since three months prior to admission with many vomiting episodes .Severity of pain had increased for one month and caused the patient to wake up at nights. The pain was not related to the position. There was also a 3-month history of poor appetite, severe fatigue, nocturnal hyperhidrosis, constipation, and noticeable weight loss (10 kg during three months).He was referred to the local hospital and was treated as dyspepsia, but symptoms did not improve.

The patient was taking prednisolone and hydroxychloroquine. He recently discontinued the hydroxychloroquine due to retinopathy. Moreover, the patient had received tamsulosin for benign prostatic hyperplasia. Also, there was a history of nephrolithiasis. He was ill in general appearance. Vital signs were within the normal range. His physical examination revealed tenderness and rebound tenderness on the right lower quadrant of the abdomen. A fixed mass 10 × 10 centimeter (cm) in diameter was palpated in the same quadrant. The rest of the systemic examination did not reveal abnormalities. We considered five differential diagnoses, including malignancy, fungal infection, lymphoma, abdominal tuberculosis and actinomycosis; according to patient history, malignancy was our initial diagnosis. Laboratory and radiologic examination were requested based on it.

Laboratory tests revealed marked increase in white blood cells(WBC), estimated sedimentation rate(ESR), C reactive protein(CRP), ferritin and decreased hemoglobin(HB). The anti-double-strand (anti-ds) DNA, complements and antinuclear antibodies(ANA) suggested that lupus was controlled. Other findings were unremarkable (Table 1) (at the end of the folder).

**Table 1:** Laboratory findings of the patient

Test	Result	Reference range
WBC (10^9^/L)	17.3	4.0–11.0
HB (g/dL)	6.8	13–16
PLT (10^3^/µL)	544	150–450
Urea (mg/dL)	28	11–55
Cr (mg/dL)	1.3	0.6–1.3
AST (U/L)	28	< 37
ALT (U/L)	11	< 41
ALP (U/L)	245	100–360
Bili T (mg/dL)	1.2	0.3–1.2
Bili D (mg/dL)	0.4	≤ 0.3
PT (S)	15	12–14
PTT	30	25–45
INR (S)	1.3	≤ 1.1
C3 (g/L)	1.3	0.88–2.01
C4 (g/L)	0.3	0.15–0.45
Anti dsDNA (IU/mL)	NEG	< 20
ANA (IU/mL)	1.3	< 20
CA125 (U/mL)	38.4	< 35
CEA (ng/mL)	< 0.5	≤ 3
AFP (ng/mL)	0.57	< 8
CA19-9 (U/mL)	< 9	0–36
Amylase (U/L)	14	< 100
Lipase (U/L)	17	< 60
CRP (mg/L)	86	0–6
ESR (mm/h)	91	0–15
LDH (IU/L)	255	120–460
Iron (µg/dL)	26	50–150
TIBC (µg/dL)	256	240–440
Ferritin (µg/dL)	1332	21–284

**Fig. 1:**
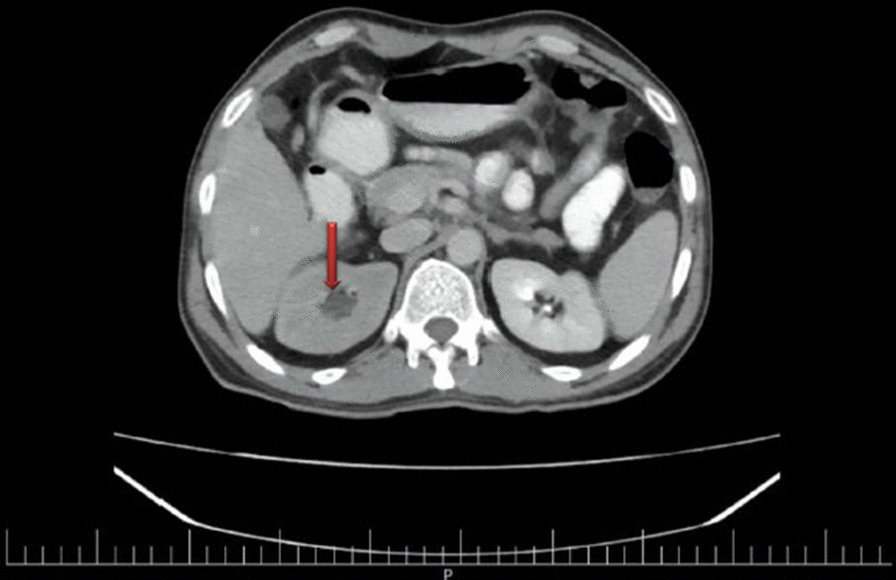
Abdominopelvic CT scan with contrast shows onset of hydronephrosis in the right kidney

Abdominopelvic computed tomography (CT) scan without contrast revealed evidence of enhancing hypoheterogenous exophytic large mass 150 × 118 × 77 mm (mm) with internal necrosis and origin of ascending colon. Also, a prolonged corticomedullary phase with moderate hydroureteronephrosis in the right kidney (Fig. 1) and one stone measuring 6 mm with two small stones in the lower pole of the left kidney were seen. Mild fluid was in the pelvic, and retroperitoneum was normal without lymphadenopathy.

Due to anemia and abdominal mass, colonoscopy was requested. Colonoscopy was done under anesthesiologist care. Anus, rectum and sigmoid were normal. The pressure effect on the sigmoid was seen. Descending colon, transverse colon, ascending colon and, cecum were normal.

**Fig. 2:**
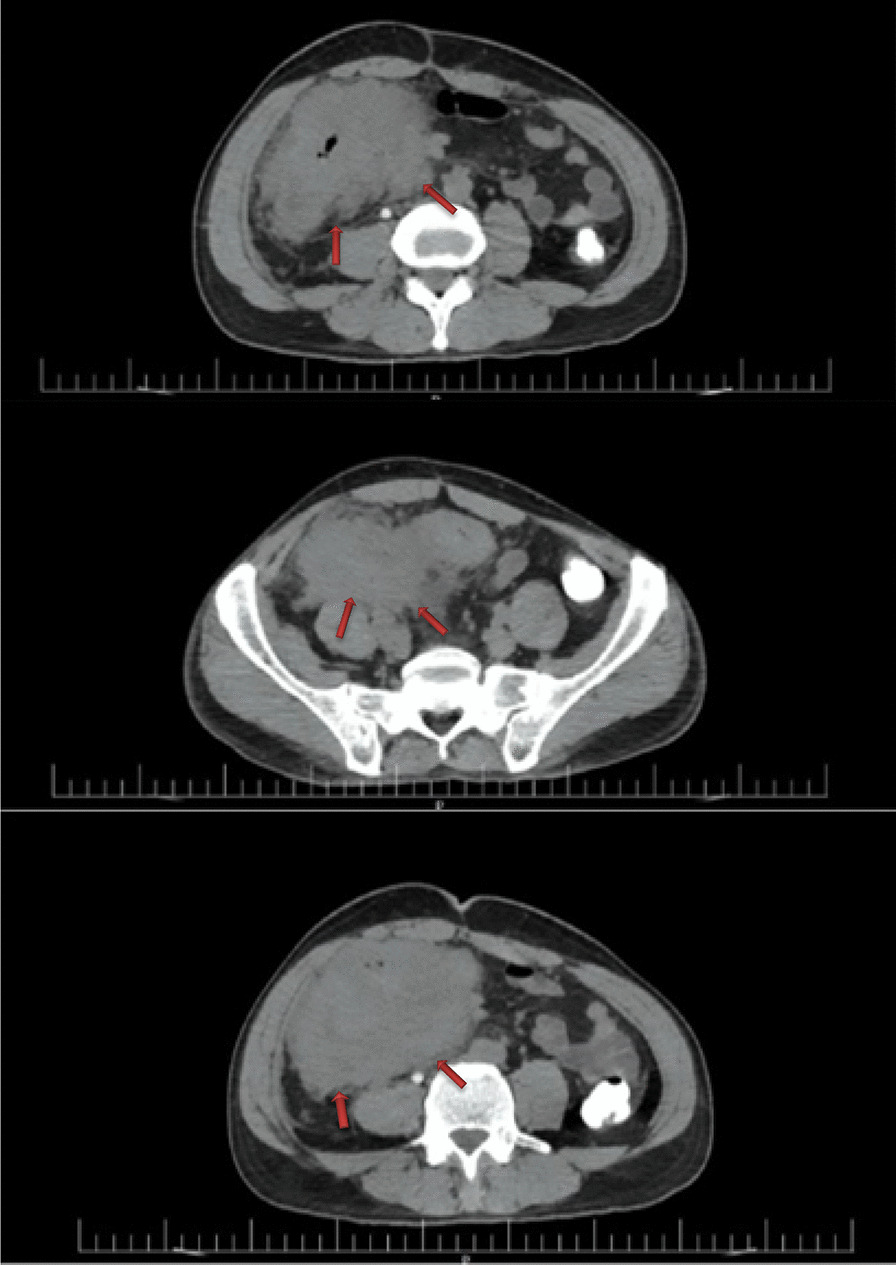
Sections of spiral abdominopelvic CT scan with contrast show a large mass surrounding with fat

Spiral abdominopelvic CT scan with contrast showed extensive wall thickening of terminal ileal loops in the right lower quadrant extending to the cecum and proximal part of ascending colon associated with surrounding fat stranding to form a large mass in the right lower quadrant (Fig. 2). Although lymphoma is prevalent in lupus patients, we did not found any lymphadenopathy or splenomegaly, so more evaluation was recommended.

The one-third distal part of the ureter invaded by mass caused mild right-side hydroureteronephrosis delayed secretion (Fig. 2). Due to acute ureteric obstruction and less likely pyelonephritis, a striated appearance was also present. Other abdominopelvic organs were normal. Ureteric double J catheter inserted for the patient.

**Fig. 3:**
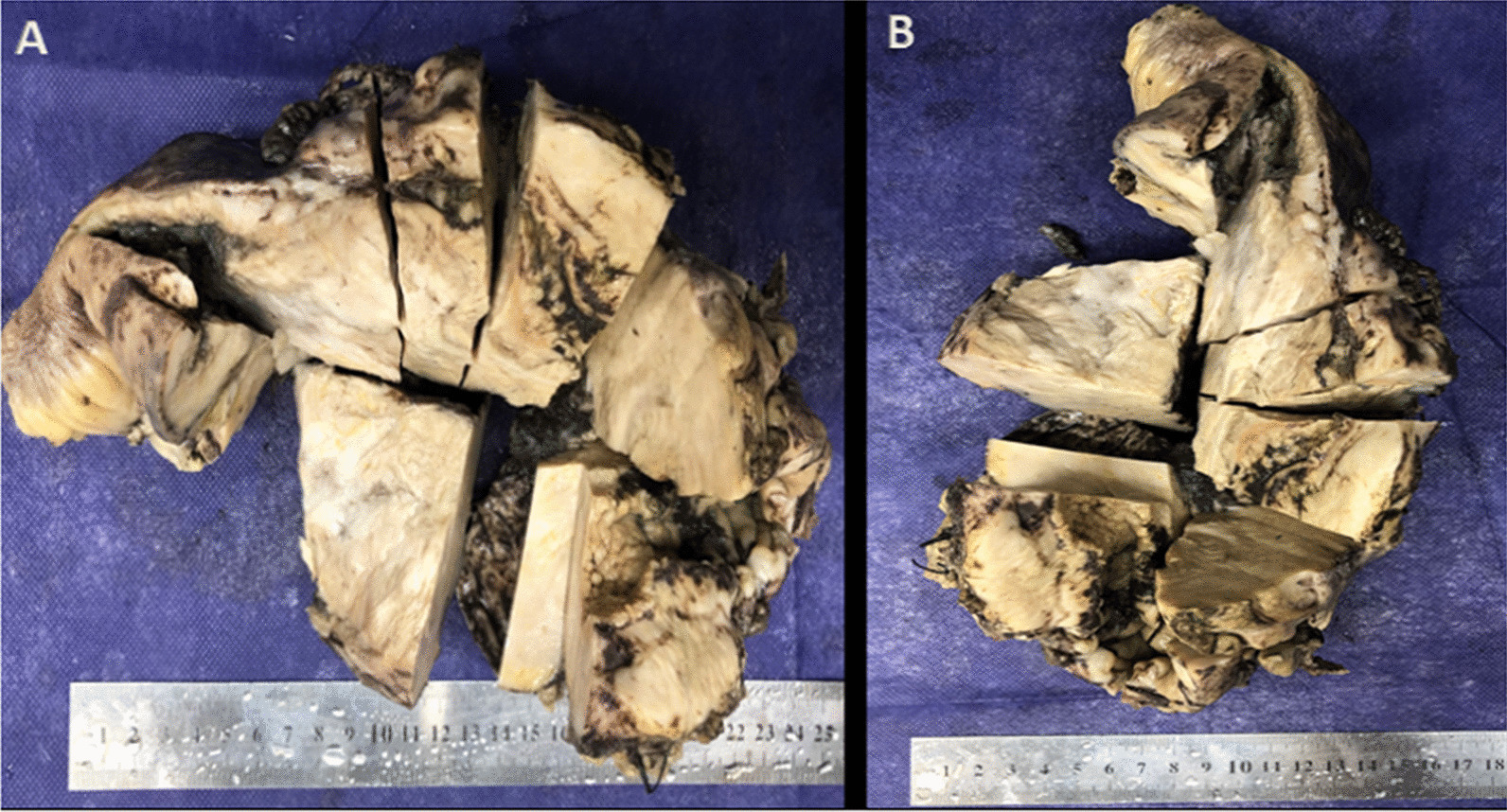
A and B shows resected mass measuring 14 *7*6.5 cm with creamy—gray color and rubbry consistency that distance from one margin is 5 cm and other margin is 2 cm

**Fig. 4:**
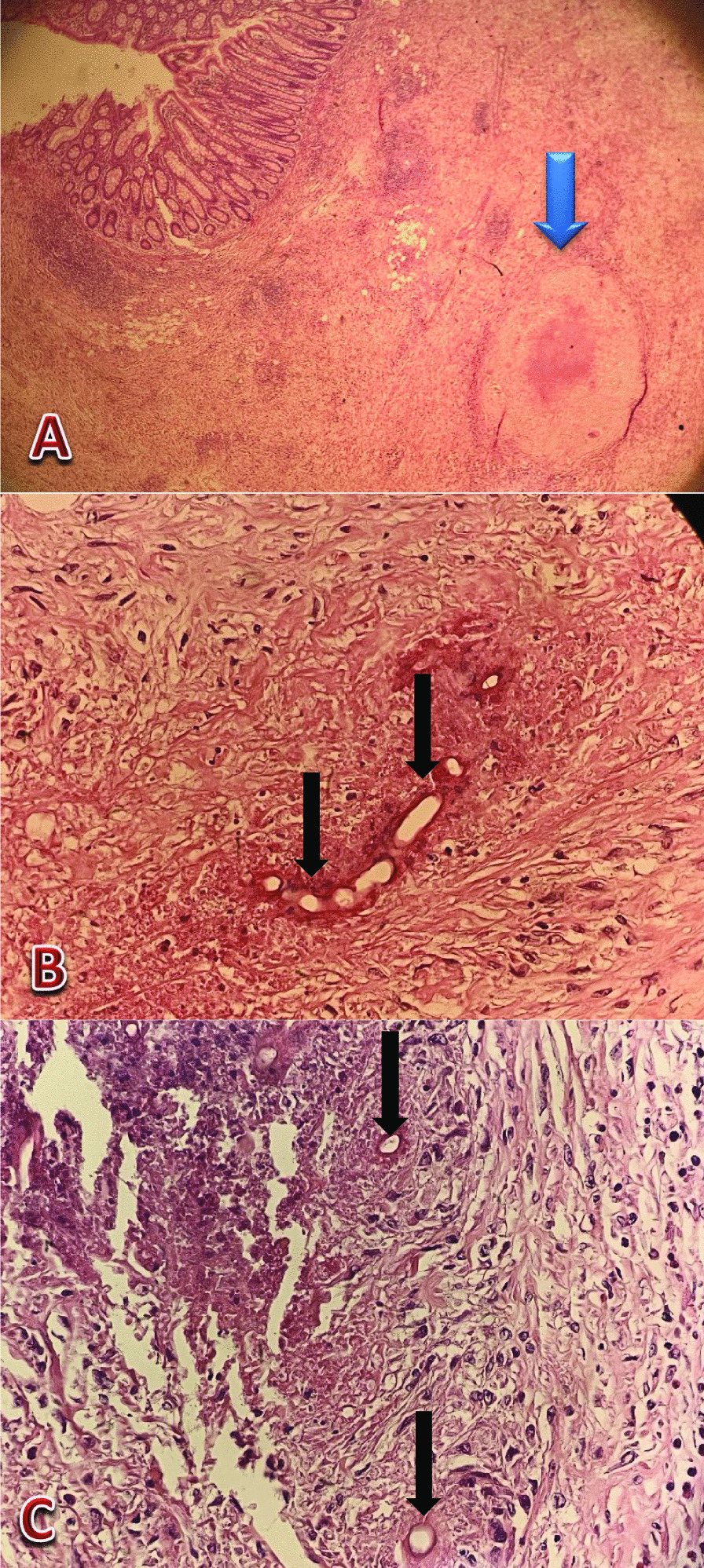
Section from bowel mass resection (**A–C**) shows severe acute and chronic inflammation with granuloma (**A**, blue arrow shows granuloma) surrounded by eosinophilic materials (splendore—hoeppli phenomenon) which are seen by hematoxylin and eosin (H&E) stain (see black arrows) (**A **× 40, **B** and **C **× 400 )

**Fig. 5:**
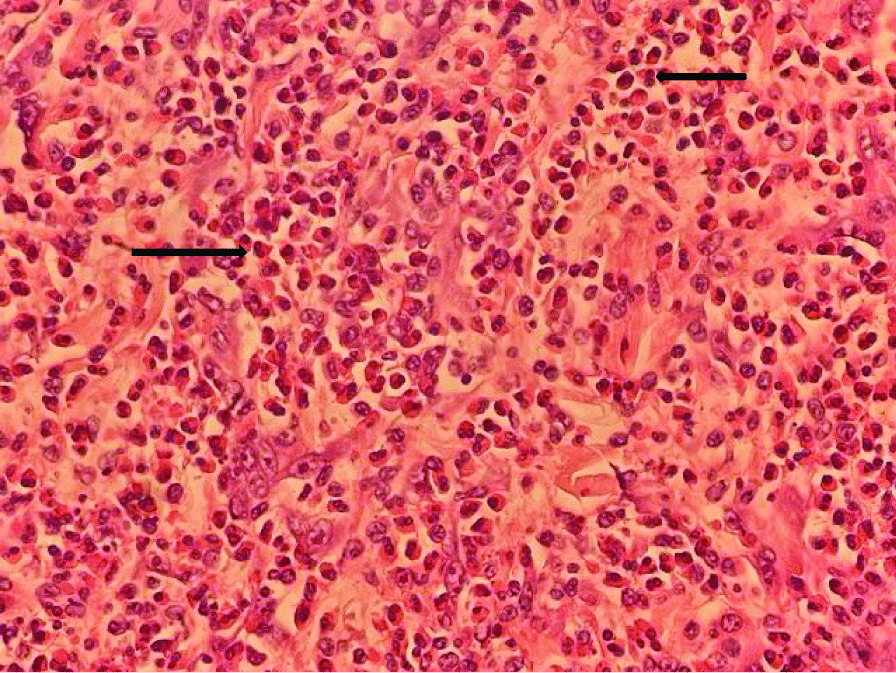
Eosinophil infiltration within the tissue is seen by H&E stain(see black arrows) (× 400)

**Fig. 6:**
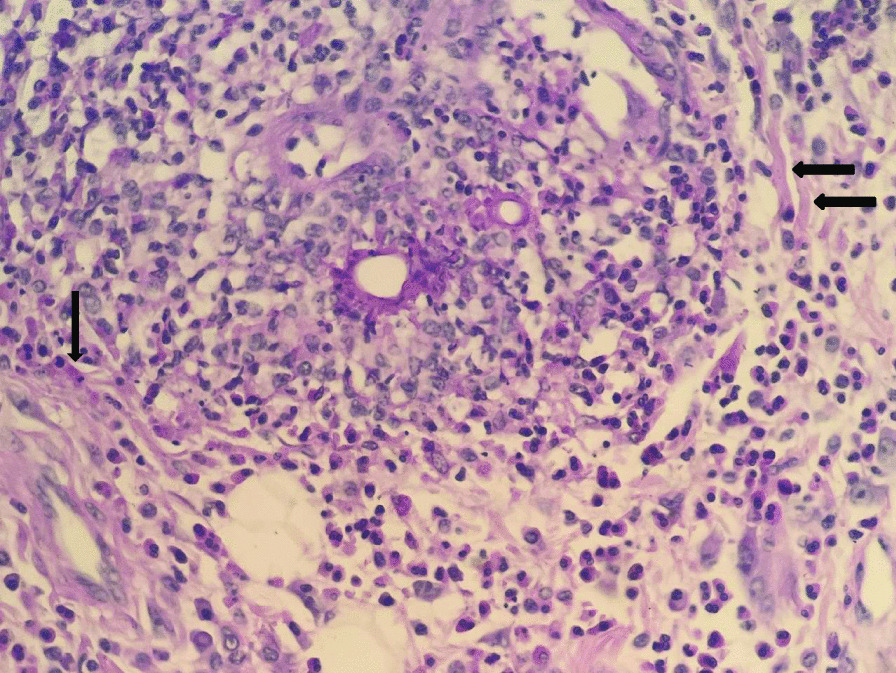
Periodic acid–Schiff (*PAS*) stain of the excised bowel. Broad septate thin walled fungal hyphae is seen (see black arrows) (× 400)

The patient underwent laparotomy. A mass was seen in the terminal ileum and ascending colon with retroperitoneal adhesion and invasion to the right ureter behind it. The mass resected with the ileum terminal and the ascending colon to the splenic flexion. The resected specimen was sent to the pathology laboratory. The specimen was cut to multiple pieces in eleven blocks and 10% of it embedded. Both unlabeled surgical resected margins were viable. Basidiobolomycosis infection was confirmed on terminal ileum and ascending colon segmental resection (Figs. 3, 4, 5, 6).

Itraconazole administered 200 mg per kilogram (mg/kg) every 12 h for 3 months and amphotericin B deoxycholate 1 mg/kg/day for 3 weeks. The patient was discharged home uneventfully and no complication was observed. The patient’s dyspepsia relieved and he gained weight.

## Discussion and conclusions

Basidiobolomycosis is a tropical fungus that primarily affects the skin. The stomach, small intestines, colon, and liver are among the major organs that can be affected [12, 13]. Nonspecific signs and symptoms make diagnosis difficult [14]. A prompt and accurate diagnosis is critical, especially when there are signs of obstruction or sepsis.

Individuals with an increased risk of basidiobolomycosis including past medical history of uncontrolled diabetes mellitus (particularly with ketoacidosis), prolonged neutropenia, prolonged corticosteroid use, hematological malignancy, organ transplant, iron overload, acquired immunodeficiency syndrome (AIDS), injection drug use and trauma/burn [12]. Furthermore, the disease has a male predilection, according to a study [2]. Our patient had risk factors including male gender and history of using corticosteroid for about eight years.

Since this fungus is an environmental saprophyte that can be found in soil and decaying vegetables and fruits, dirt or feces ingestion, or food contaminated by either, appear to be the routes of infection [15]. Our patient had been in contact with soil constantly due to his job as a farmer which could be the source of infection.

Considering that in previous case report and review studies patients were mostly children about one to ten years old, so pediatricians might consider it among their differential diagnosis [16–18].

The Patient’s general symptoms like abdominal pain, anorexia, constipation, and weight loss were similar to other reported cases but the patient had no bleeding although GI bleeding does not show a predilection for any particular age group and has been reported in patients aged 1.5 years to 80 years in most of the cases [12–14]. The colonoscopy in our case was mostly normal. There was only a pressure effect on the sigmoid.

Pathologic features of basidiobolomycosis include presence of splendore-hoeppli bodies and numerous eosinophils, as well as intensely radiating eosinophilic granular material surrounding the fungal elements [19]. Our pathological findings were in concordance with it.

In our case basidiobolomycosis was confirmed microscopically, though molecular testing for basidiobolomycosis may prove to be the most accurate method of diagnosis. Ribosomal DNA sequencing can precisely confirm the diagnosis of infection in formalin-fixed paraffin-embedded (FFPE) intestinal tissue [20]. Moreover, molecular testing using polymerase chain reaction (PCR) assays with panfungal specific primers that amplified the internal transcribed spacer 1 and 2 regions of ribosomal DNA and sequence analysis of the PCR products using the basic local alignment search tool can also confirm the disease [13].

The gold standard of GIB definite diagnosis is culture [21, 22], but the patient underwent surgery with the preoperative diagnosis of mass. As a result, the specimen was sent to a lab in formalin and no culture was done. However, the histopathology was distinctive enough.

Although the antifungal treatment alone has been described to be effective, treatment is often presumptive because it is difficult to establish a definitive diagnosis [14].

To prevent recurrence, a wide margin radical resection should be used. literatures supports the use of early surgical intervention to reduce mortality [2, 15, 22–24].

Our patient underwent laparotomy and was prescribed itraconazole and amphotericin B deoxycholate. Lipid formulation of amphotericin B is preferred due to its low renal toxicity [12], however in our case we did not have access to liposomal amphotericin B.

In conclusion, fungal infection should be among the differential diagnoses for adults taking corticosteroid and presenting abdominal mass. Raising awareness of this condition among infectious disease specialists, and pathologists especially in endemic regions and developing world countries may lead to the discovery of more cases, allowing doctors to make diagnosis earlier and manage the case appropriately.

## Data Availability

The data sets used during the current study are available from the corresponding author on reasonable request.

## Abbreviations

GI: Gastrointestinal
GIB: Gastrointestinal basidiobolomycosis
CT: Computed tomography
PAS: Periodic acid–Schiff
H&E: Hematoxylin and eosin
FFPE: Formalin-fixed paraffin-embedded
PCR: Polymerase chain reaction
